# Obstetric emergencies and adverse maternal-perinatal outcomes in Ethiopia; A systematic review and meta-analysis

**DOI:** 10.3389/fgwh.2022.942668

**Published:** 2022-10-26

**Authors:** Masresha Leta, Nega Assefa, Maleda Tefera

**Affiliations:** ^1^Harar Health Science College, Harar, Ethiopia; ^2^School of Nursing and Midwifery, College of Health and Medical Sciences, Haramaya University, Harar, Ethiopia

**Keywords:** adverse outcome, perinatal death, maternal death, adverse, obstetrics emergency

## Abstract

**Background:**

Obstetric emergencies are life-threatening medical problems that develop during pregnancy, labor, or delivery. There are a number of pregnancy-related illnesses and disorders that can endanger both the mother's and the child's health. During active labor and after delivery, obstetrical crises can arise (postpartum). While the vast majority of pregnancies and births proceed without a hitch, all pregnancies are not without risk. Pregnancy can bring joy and excitement, but it can also bring anxiety and concern. Preterm birth, stillbirth, and low birth weight are all adverse pregnancy outcomes, leading causes of infant illness, mortality, and long-term physical and psychological disorders.

**Purpose:**

The purpose of this study is to assess the magnitude and association of obstetric emergencies and adverse maternal-perinatal outcomes in Ethiopia.

**Method:**

We used four databases to locate the article: PUBMED, HINARI, SCIENCE DIRECT, and Google Scholar. Afterward, a search of the reference lists of the identified studies was done to retrieve additional articles. For this review, the PEO (population, exposure, and outcomes) search strategy was used. Population: women who had obstetric emergencies in Ethiopia. Exposure: predictors of obstetric emergencies. Outcome: Women who had an adverse perinatal outcome. Ethiopian women were the object of interest. The primary outcome was the prevalence of adverse maternal and perinatal outcomes among Ethiopian women. Obstetrical emergencies are life-threatening obstetrical conditions that occur during pregnancy or during or after labor and delivery. The Joanna Briggs Institute quality assessment tool was used to critically appraise the methodological quality of studies. Two authors abstracted the data by study year, study design, sample size, data collection method, and study outcome. Individual studies were synthesized using comprehensive meta-analysis software and STATA version 16. Statistical heterogeneity was checked using the Cochran Q test, and its level was quantified using the *I*^2^ statistics. Summary statistics (pooled effect sizes) in an odd ratio with 95% confidence intervals were calculated.

**Result:**

A total of 35 studies were used for determining the pooled prevalence of adverse maternal and perinatal outcomes; twenty-seven were included in determining the odd with 95% CI in the meta-analysis, from which 14 were cross-sectional, nine were unmatched case-control studies, and 14 were conducted in the south nation and nationality Peoples' Region, and eight were from Amhara regional states, including 40,139 women who had an obstetric emergency. The magnitude of adverse maternal and perinatal outcomes following obstetric emergencies in Ethiopia was 15.9 and 37.1%, respectively. The adverse maternal outcome increased by 95% in women having obstetric emergencies (OR 2.29,95% CI 2.43–3.52), and perinatal deaths also increased by 95% in women having obstetric emergencies (OR 3.84,95% CI 3.03–4.65) as compared with normotensive women.

**Conclusion:**

This review demonstrated the high prevalence of perinatal mortality among pregnant women with one of the obstetric emergencies in Ethiopia. Adverse maternal and perinatal outcomes following obstetric emergencies such as ICU admission, development of PPH, giving birth *via* CS, maternal death, NICU admission, LBW, and perinatal death were commonly reported in this study.

## Introduction

Obstetric emergencies are life-threatening medical problems that develop during pregnancy, labor, or delivery. There are a number of pregnancy-related illnesses and disorders that can endanger both the mother's and the child's health. Obstetrical emergencies may also occur during active labor and after delivery (postpartum) (1).

While the vast majority of pregnancies and births proceed without a hitch, all pregnancies are not without risk. Pregnancy can be a time of happiness and anticipation, but it can also be a period of anxiety and concern (2).

For a smooth transition from life in the womb to life outside the womb, a delicate balance between the mother's health, the course of the pregnancy, the delivery method, and early postoperative care is essential. Pregnancy and the neonatal period significantly impact maternal and child survival outcomes (3).

Adverse pregnancy outcomes are those pregnancy outcomes other than normal live birth, which mostly include preterm birth, stillbirth, and low birth weight, which are the major causes of neonatal morbidity, mortality, and long-term physical and psychological problems (4).

In both developing and developed countries, adverse birth outcomes such as prematurity, low birth weight, and stillbirth are major issues. Around 15 million babies are born prematurely worldwide, accounting for more than one in every ten births. More than one million newborns die soon after birth, and countless others are left with lifelong physical, neurological, or educational problems, frequently at a high cost to families and societies. Moreover, women also die as a result of complications during and after pregnancy and childbirth (5).

The majority of these problems emerge during pregnancy and can be avoided or treated. Other problems may be present before pregnancy, but they will increase during the pregnancy if they are not treated as part of the woman's therapy. Severe bleeding (typically after childbirth), infections (usually after childbirth), high blood pressure throughout pregnancy (pre-eclampsia and eclampsia), complications following delivery, and unsafe abortion are the leading causes of maternal death (6). As a result, the purpose of this study is to determine the magnitude of adverse maternal and perinatal outcomes following an obstetric emergency, as well as to describe the relationship between an obstetric emergency and adverse maternal-perinatal outcomes in women who have obstetric emergencies during pregnancy or labor.

## Methods

The review flow was based on the established Preferred Reporting Items for Systematic Reviews and Meta-Analyses (PRISMA) reporting guidelines.

### Inclusion criteria

The participants in this study were pregnant mothers who had an obstetric emergency and had a poor maternal-perinatal outcome. The studies were published in English from January 2010 to March 2022.

All published and unpublished observational (cross-sectional, case-control, prospective, and retrospective cohort studies) studies conducted in Ethiopia were included in this search.

Pregnant women who had obstetric emergencies were the study subjects, and the consequence was a negative maternal-perinatal outcome.

### Data source, study selection, and search strategy

We used four databases to locate the article: PUBMED, HINARI, SCIENCE DIRECT, and Google Scholar. Onwards, a search of the reference lists of the identified studies was done to retrieve additional articles. For this review, the PEO (population, exposure, and outcomes) search strategy was used. Population: women who had obstetric emergencies in Ethiopia. Exposure: predictors of an obstetric emergency. Outcome: women who had adverse perinatal outcomes. Ethiopian women were the object of interest. The primary outcome was the prevalence of adverse maternal and perinatal outcomes among Ethiopian women. Obstetrical emergencies are life-threatening conditions that occur during pregnancy or after labor (7).

The secondary outcomes were: the predictors of obstetric emergencies such as maternal age, place of residence, ANC visit, gravidity, etc. For each selected PEO component, the electronic databases were searched using keywords and the medical subject heading [MeSH] terms. The keyword was “emergency obstetric OR PIH OR APH OR obstructed labor OR premature rupture of membranes (PROM) OR prolapsed umbilical cord OR placenta accreta OR rupture of the uterus OR amniotic fluid embolism AND adverse maternal outcomes OR ICU admission OR PPH OR hysterectomy OR distractive delivery OR CS OR long hospital stay OR blood transfusion OR maternal death OR adverse perinatal outcomes OR NICU admission OR birth asphyxia OR low APGAR score OR low birth weight OR premature birth OR RDS OR long hospital stay OR stillbirth OR neonatal death.” To address unpublished literature, the searches include literature from Google and Google scholar. The search was limited to papers published in English from January 2010 to March 2022 in Ethiopia.

### Study selection

**Figure 1:**
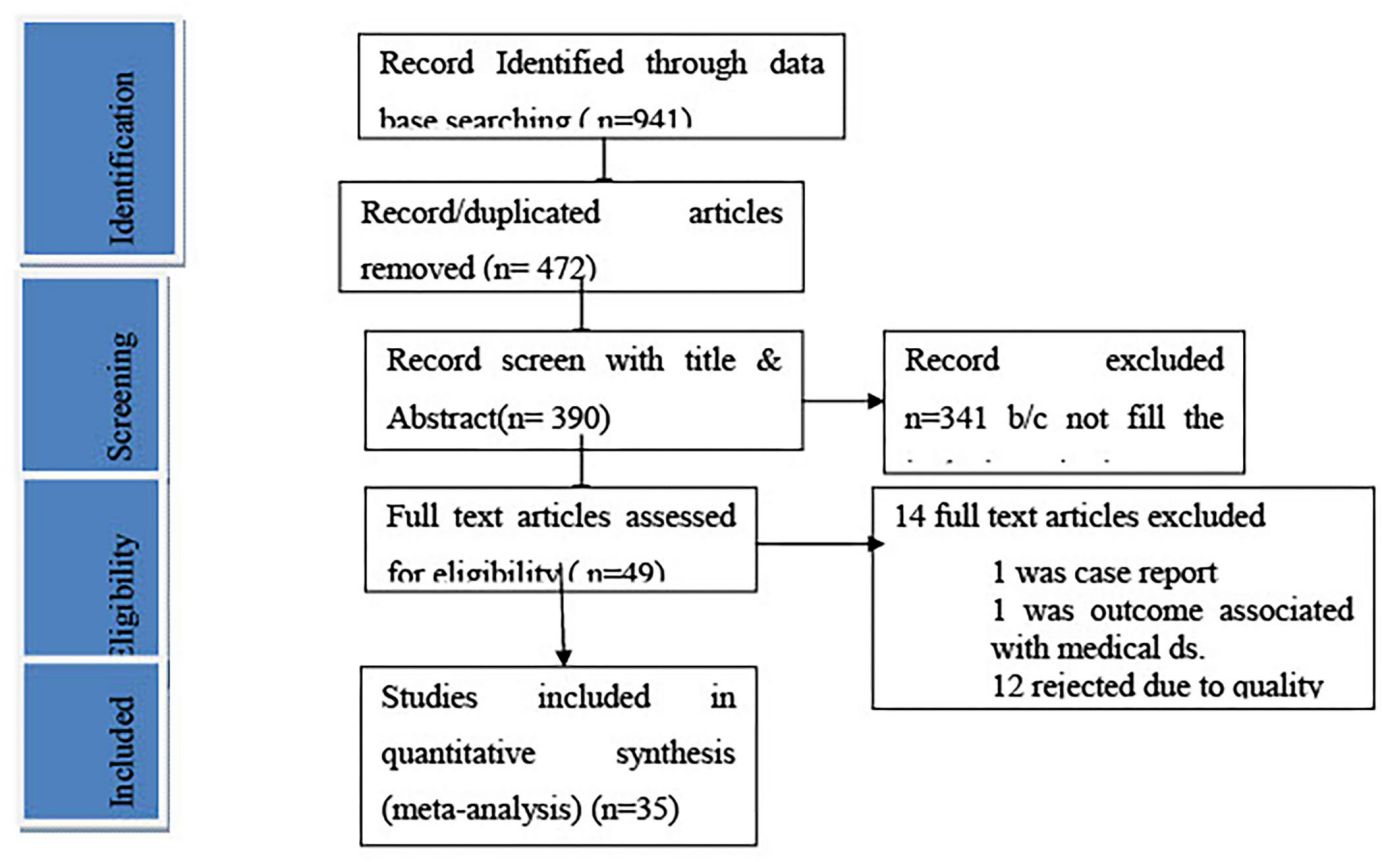
PRISMA flow chart.

We include all observational studies published in English that show the relationship between obstetric emergencies and adverse maternal or perinatal outcomes, which were done in primary, general, or referral facilities, by excluding community-based studies, case series, and case report studies. The studies conducted outside of Ethiopia do not consider the WHO definition of an obstetric emergency. Studies on adverse outcomes with medical diseases and studies describe neither maternal nor perinatal outcomes. Thirty-two studies were finally identified, and the detailed data searching strategy was presented in the PRISMA flowchart (Figure 1).

### Study selection procedure: Screening

In two phases, two authors (M.L. and N.A.) independently assessed the eligibility of chosen studies. To determine eligibility for the second step, which comprises full-text screening, the titles and abstracts of the retrieved literature were first assessed. During this phase, the authors conducted additional screening to determine whether or not the participants were eligible to participate in the study. Disagreements between the two reviewers were resolved by a conversation with the third review author and/or by consensus (M.T).

### Data collection process

The data were retrieved from the eligible studies by the first review author (M.L.), which were then kept on a data extraction sheet and reviewed by the second review author (N.A.). Disagreements between the two reviewers were settled through conversation; otherwise, the third review author (M.T.) made the final decision. Based on the authors' names, sample size, outcomes, and study location, the studies were examined for duplication. Data were extracted from the year of the study, the region in which it was conducted, the study design, the type of obstetric emergency, the sample size, and the extent of the unfavorable outcome. During data extraction, adverse maternal outcomes (destructive delivery, CS, extended hospital stay, and maternal mortality) are classified as maternal outcomes. In contrast, perinatal outcomes (stillbirth, NICU admission, sepsis, and low birth weight) are classified as perinatal outcomes.

### Assessment of methodological quality

The authors independently assessed all included studies for methodological validity using the JBI (Joanna Briggs Institute). The inclusion criteria, the randomness of participant selection, identification of research participants, the precision of measurement of outcomes of interest, use of proper statistical analysis methods, and documentation of sources of bias or confounding were all given special attention.

### Statistical analysis

Using studies that reported pregnancy outcomes, an unadjusted odds ratio (OR) with a 95% confidence interval (CI) was calculated using both STATA version 16 (for adverse perinatal outcome) and comprehensive meta-analysis version 2 package (for adverse maternal outcome). In addition to the overall pooled prevalence of perinatal death, subgroup and sensitivity analyses were also performed. Considering the “synthesis of results,” the raw proportion was transformed into a suitable unit for meta-analysis using the logit transformation. To produce an overall summary estimate of the prevalence across studies and pooled study-specific estimates, a random effects model was employed to account for probable heterogeneity between studies, which defaults to the fixed effects model method in the absence of heterogeneity (8). The meta-analysis results were graphically shown using forest plots. The Chi^2^ test (Cochran Q test) was used to examine for statistical heterogeneity with a p-value of 0.05. The *I*^2^ statistic/s were used to determine the degree of heterogeneity among the studies, with an *I*^2^ value of <50% implying substantial heterogeneity. An obstetric emergency can be considered an exposure throughout the study, while unfavorable maternal and perinatal outcomes can be considered an outcome.

### Risk of bias across studies

The study assessed the possibility of publication bias using Egger's linear regression test as a formal statistical test for publication bias (9). The Egger's test resulted in a *P*-value of <0.10, indicating statistically significant publication bias.

### Additional analyses

Subgroup analysis was carried out based on publication year, study design, and Ethiopian regions. Furthermore, the sensitivity analysis investigated the extent to which modifications in the techniques or data utilized in specific studies influenced the overall findings of a review. The outliers and inliers were also considered in the sensitivity analysis.

## Results

### Description of the studies

We found 941 studies through searching the electronic medical database and other essential sources. Of those identified studies, 472 articles were removed due to duplication, and the remaining 390 articles screened by topic and abstract of these 341 studies were excluded because the content in the title and abstract did not match with the recent study. The remaining 49 full-text studies were reviewed to assess eligibility, and 14 studies were excluded due to duplication, an inconsistent study outcome, and a different study population from our study. The last 35 studies were critically appraised, and the studies that got higher scores were included in the study: 14 cross-sectional, nine unmatched case-control, five retrospective, five case-control, and two prospective cohorts.

Almost all studies adjusted for confounding variables, such as adverse outcomes resulting from other than obstetric emergencies and twin pregnancies.

### Study characteristics

The characteristics of the cross-sectional [1,024] prospective or retrospective cohort [2,531] and case-control studies [3,244] included in this review from 6 regional states of Ethiopia are summarized in Table 1. The majority of studies were conducted in SNNPR, Amhara, and Oromiya regional states, which are 14, 8, and 6, respectively.

**Table 1:** Characteristics of included studies in Ethiopia.

**References**	**Study design**	**Region**	**Type of obstetric emergency**	**Sample size**	**Adverse maternal outcome**	**Adverse perinatal outcome**
Astatikie et al. (10)	Cross-sectional	Amhara	Uterine rupture	242	202	238
Jaleta et al. (11)	Retrospective cohort	Oromiya	HDP	259	170	241
Shimelis et al. (12)	Cross-sectional	Oromiya	Obstructed Labor	179	176	92
Getaneh et al. (34)	Retrospective cohort	Amhara	Eclampsia	251	139	98
Adere et al. (35)	Unmatched case-control	SNNPR	Placenta Preavia	303	285	246
Getachew et al. (13)	Cross-sectional	Oromiya	Obstructed Labor	277	125	149
Getachew et al. (13)	Case-control	SNNPR	PIH	69	27	25
Berhe et al. (36)	Prospective cohort	Tigray	PIH	260	52	168
Belay Tolu et al. (37)	Prospective	Addis Abeba	Pre-eclampsia	164	17	132
Melese et al. (38)	Cross-sectional	Amhara	PIH	456	299	223
Endeshaw and Berhan (27)	Retrospective cohort	SNNPR	HDP	1,015	51	583
Dadi et al. (39)	Case-control	SNNPR	Uterine rupture	363	154	107
Adane et al. (14)	Cross-sectional	Amhara	APH, PIH	481	104	22
Legu et al. (45)	Case-control	SNNPR	APH, PIH	434	73	122
Abdo et al. (46)	Cross-sectional	SNNPR	Pre-eclampsia, PROM	304	62	18
Bereka et al. (44)	Case-control	Tigray	Uterine rupture	336	43	309
Degno et al. (15)	Cross-sectional	Oromiya	PROM	580	110	23
Asseffa and Demissie (40)	Retrospective	SNNPR	HDP	7,347	42	49
Abebe et al. (16)	Unmatched case-control	SNNPR	HDP	407	0	86
Gizaw et al. (17)	Case-control	Oromiya	PIH	342	0	17
Astatikie et al. (10)	Cross-sectional	Amhara	Uterine rupture	10,379	192	238
Deressa et al. (23)	Cross-sectional	Addis-abeba	PIH	384	0	13
Gejo et al. (18)	Unmatched Case-control	SNNPR	PIH, PROM	213	0	55
Eyeberu et al. (41)	Cross-sectional	Harari	APH, PIH	834	0	84
Tasew et al. (19)	Unmatched case-control	Tigray	APH	264	0	11
Fikre et al. (14)	Case-control	SNNPR	APH Obstructed Labor	424	0	154
Welegebriel et al. (20)	Case-control	SNNPR	Uterine rupture, APH	540	0	94
Wondimu et al. (42)	Cross-sectional	Oromiya	PIH	260	0	11
Alemu et al. (21)	Unmatched case-control	Amhara	PIH PROM	390	0	99
Vata et al. (22)	Retrospective	SNNPR	Pre-eclampsia	7,702	178	40
Deressa et al. (23)	Cross-sectional	Addis Abeba	PIH	3,732	0	13
Wosenu et al. (24)	Case-control	Amhara	Meconium-stained Amniotic fluid	273	49	58
Mamo et al. (25)	Cross-sectional	SNNPR	APH, PROM	311	0	192
Assefa et al. (43)	Cross-sectional	Amhara	APH.PIH, meconium stained	364	0	62
Mihiretu et al. (26)	Cross-sectional	SNNPR	Obstructed labor, cord prolapse, preeclampsia	300		34
Total				40,139	2,501	4,106

The sample size of the studies ranged from 69 in SNNPR to 10,379 in Amhara regional state, 38 with a total of 40,139 participants. Overall, 2,501 pregnant women and 4,106 infants were recorded as suffering from disorders due to obstetric emergencies during the antepartum and/or intrapartum and/or postpartum period.

### Association between obstetric emergencies and adverse maternal outcome

Sixteen studies have reported a significant relationship between the exposure variable and the intended outcome, and the magnitude of maternal mortality ranges from 0.1 to 8%. From these, two studies described the association between obstetric emergencies and maternal death. 12.8 (2.99, 54.49) (27) and 2.83 (1.3, 6.15) (13). According to the reviewed studies, maternal mortality is 3–13 times more prevalent in women having obstetric emergencies than in normotensive women (Table 2).

**Table 2:** Obstetric emergencies and maternal mortality.

**No**	**Author**	**Region**	**Study design**	**Sample size**	**Type of obstetric emergency**	**Magnitude**	**OR**
1	Jaleta et al. (11)	Oromiya	Retrospective	259	Uterine rupture	2.3%	
2	Getaneh et al. (34)	Amhara	Retrospective	251	Eclampsia	7.1%	
3	Belay Tolu et al. (37)	Addis Abeba	Prospective	164	Pre-eclampsia	1.2%	
4	Bereka et al. (44)	Tigray	Case-control	336	Uterine rupture	0.2%	
5	Dadi et al. (39)	SNNPR	Case-control	363	Utrine rupture	3%	
6	Asseffa and Demissie (40)	SNNPR	Retrospective	7,347	HDP	0.1%	
7	Astatikie et al. (10)	Amhara	Cross-sectional	10,379	Uterine rupture	0.15%	
8	Endeshaw and Berhan (27)	SNNPR	Retrospective	1,015	HDP	5.02%	12.8(2.99,54.49)
9	Getachew et al. (13)	SNNPR	Unmatched case-control	207	PIH	3.04%	2.83(1.3,6.15)

### Association between obstetric emergencies and adverse perinatal outcome

Twenty-two studies have reported a significant relationship between obstetric emergencies and adverse perinatal outcomes. Of those studies, 11 showed the association of obstetric emergency with adverse outcomes of stillbirth, six studies with EOND, five studies with NICU admission, seven studies with preterm birth, and two with perinatal deaths. Six studies indicated the occurrence of EOND in relation to obstetric emergencies.

According to reviewed studies, the magnitude of EOND ranges from 0.2 to 19%. The risk of EOND is 1.5–12 times more likely to occur in women having obstetric emergencies than in normotensive women. Eleven studies also examined the onset of stillbirth with maternal obstetric complications. The magnitude of stillbirth ranged from 0.027 to 81% in women with obstetric emergency problems. The risk of stillbirth is 2.35–24 times more likely to occur in women with obstetric emergency problems than in normotensive women. In addition to this, seven studies indicated that the association of preterm birth with obstetric emergencies and the magnitude of preterm birth in Ethiopia ranged from 3.2 to 50% in women having obstetric complications, as well as the risk of preterm birth was increased by 0.182–40% in women having obstetric complications than normotensive women. Nevertheless, five studies were reviewed and showed a significant association between obstetric emergencies and the rate of NICU admission. The magnitude of NICU admission is 0.2–31 times higher in neonates delivered from mothers having obstetric emergency problems, and the risk of being admitted to NICU is 2–34 times higher in neonates born from mothers with obstetric complications.

### The magnitude of adverse maternal and perinatal outcome

**Figure 2:**
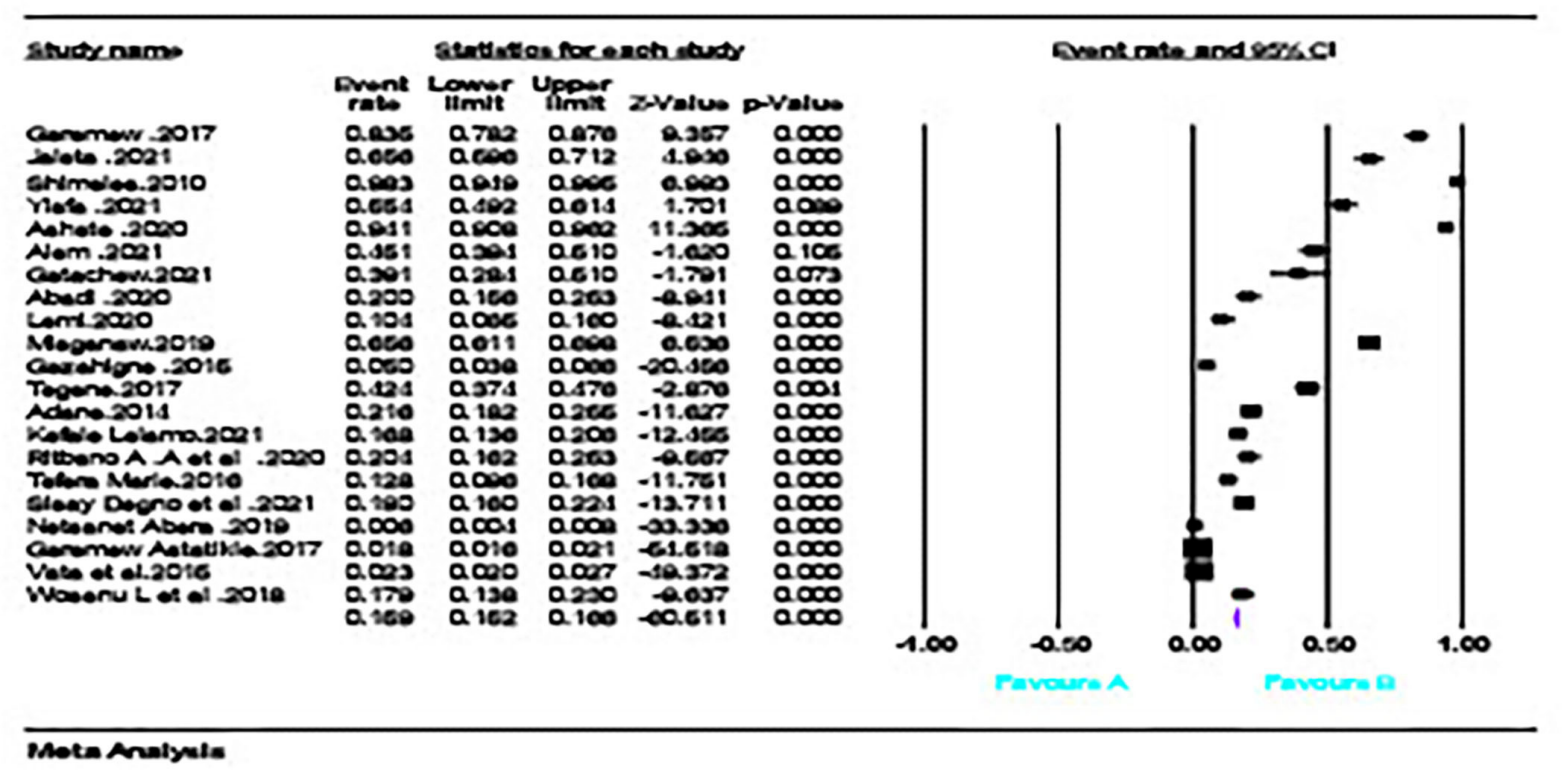
Magnitude of adverse maternal outcome following obstetrics emergency.

**Figure 3:**
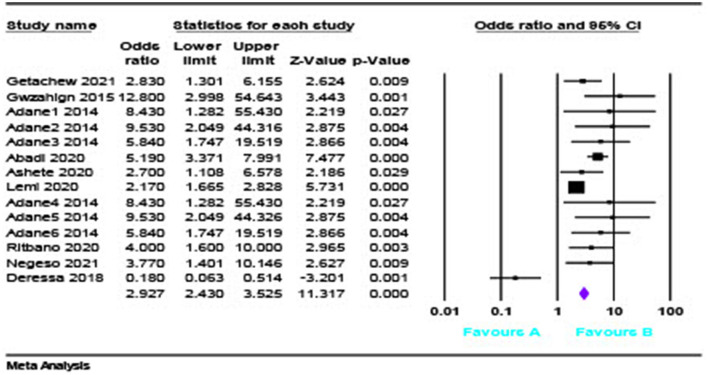
Magnitude of adverse perinatal outcome following obstetrics emergency.

The pooled prevalence of adverse maternal outcomes following obstetric emergencies in Ethiopia was 15.9% (95% CI = [0.152, 0.166]); (Figure 2), whereas the pooled prevalence of adverse perinatal outcomes following obstetric emergencies in Ethiopia was 37.1% (95% CI = [0.362, 0.381]) (Figure 3).

### Obstetric emergencies and adverse maternal outcomes

**Figure 4:**
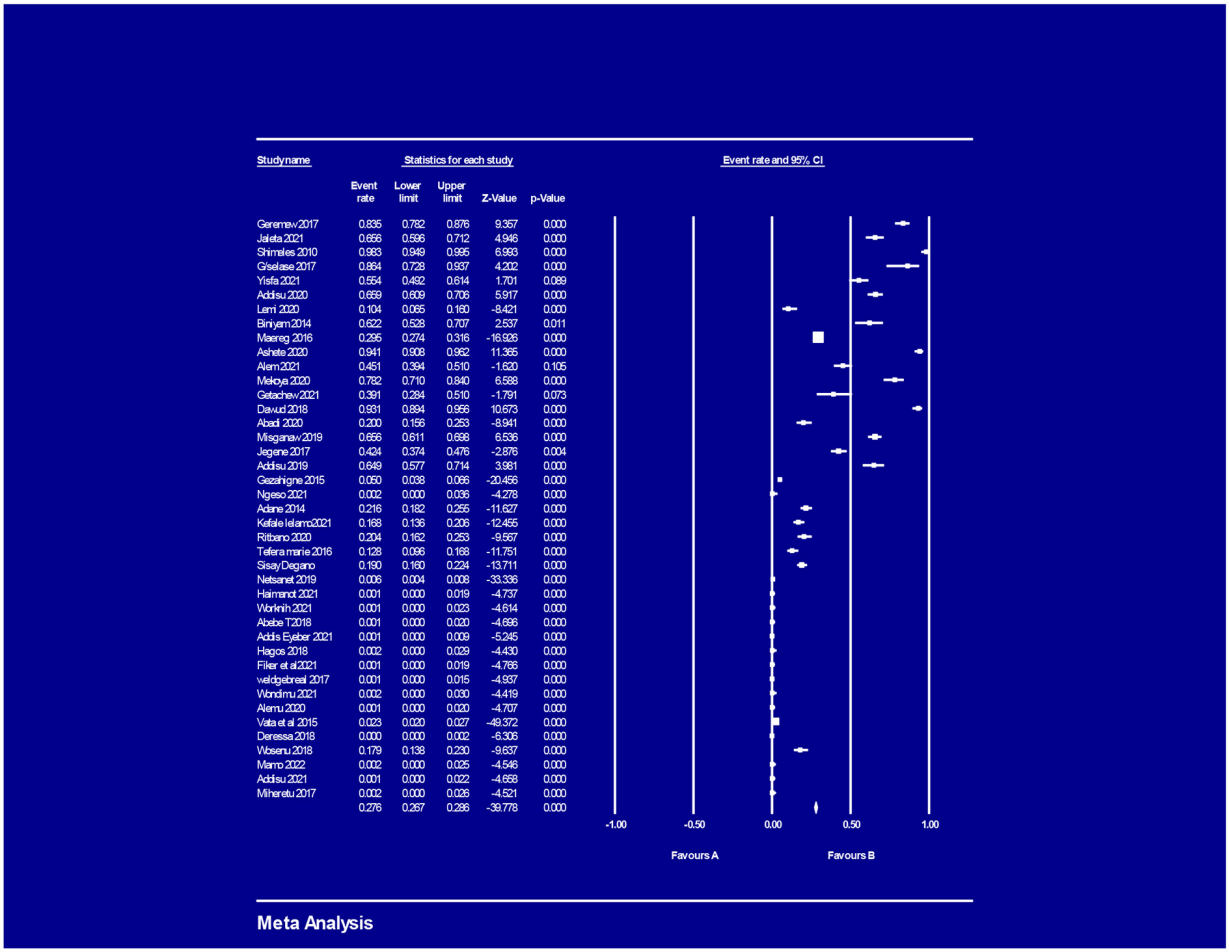
The pooled analysis of the risk of adverse maternal outcome.

The pooled analysis showed that the risk of adverse maternal outcome was 2.92 times OR = 2.927(95% CI = [2.43, 3.52]) (Figure 4) more likely to occur in women having obstetric emergencies than normotensive women.

### Publication bias

**Figure 5:**
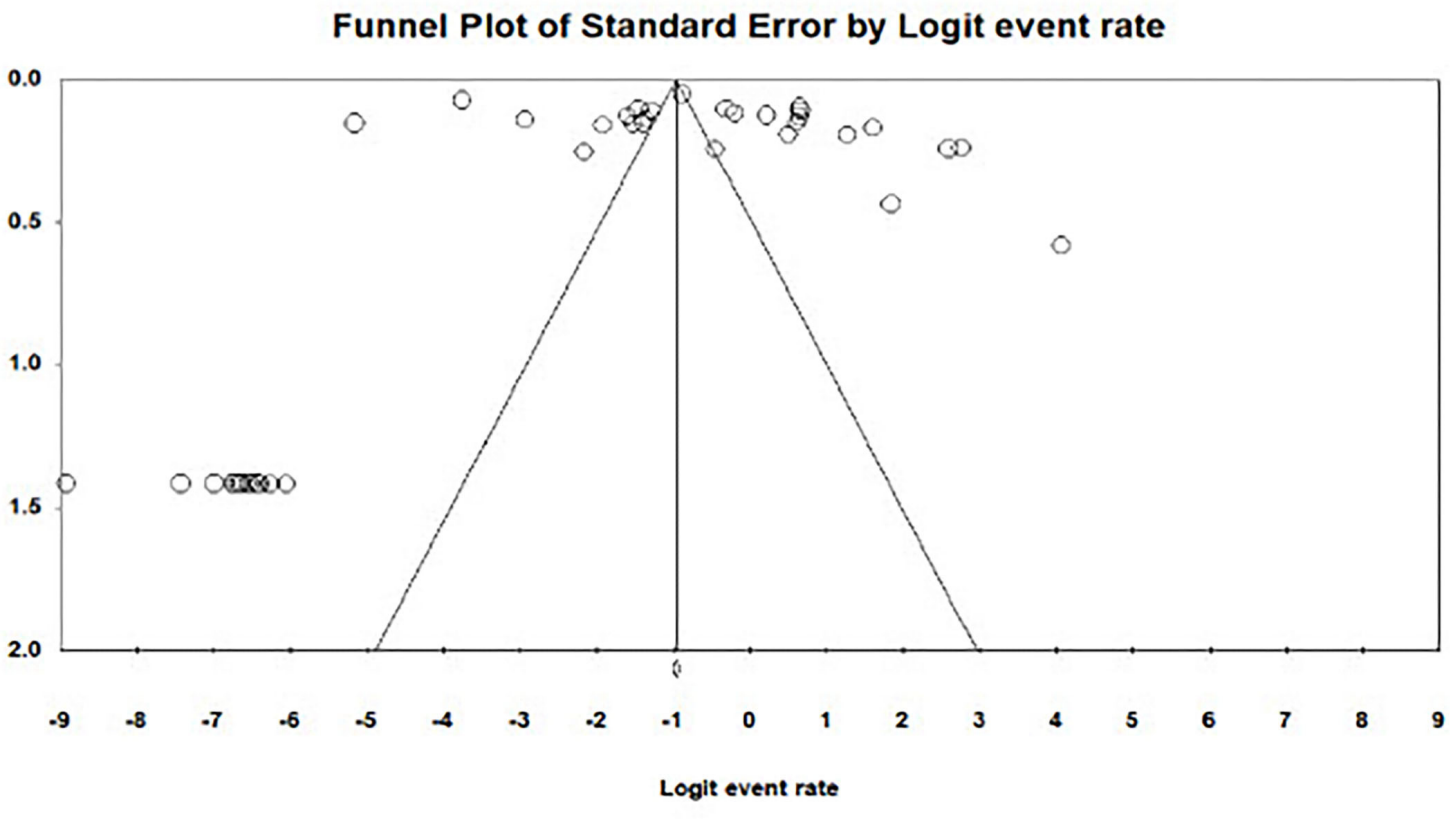
Publication bias for the adverse maternal outcome.

There was evidence of publication bias across studies reporting on adverse maternal outcomes following obstetric emergencies. Egger's test for bias gave a *p*-value of 0.41 and a funnel plot of asymmetry in shape (Figure 5).

### Obstetric emergencies and perinatal deaths

**Figure 6:**
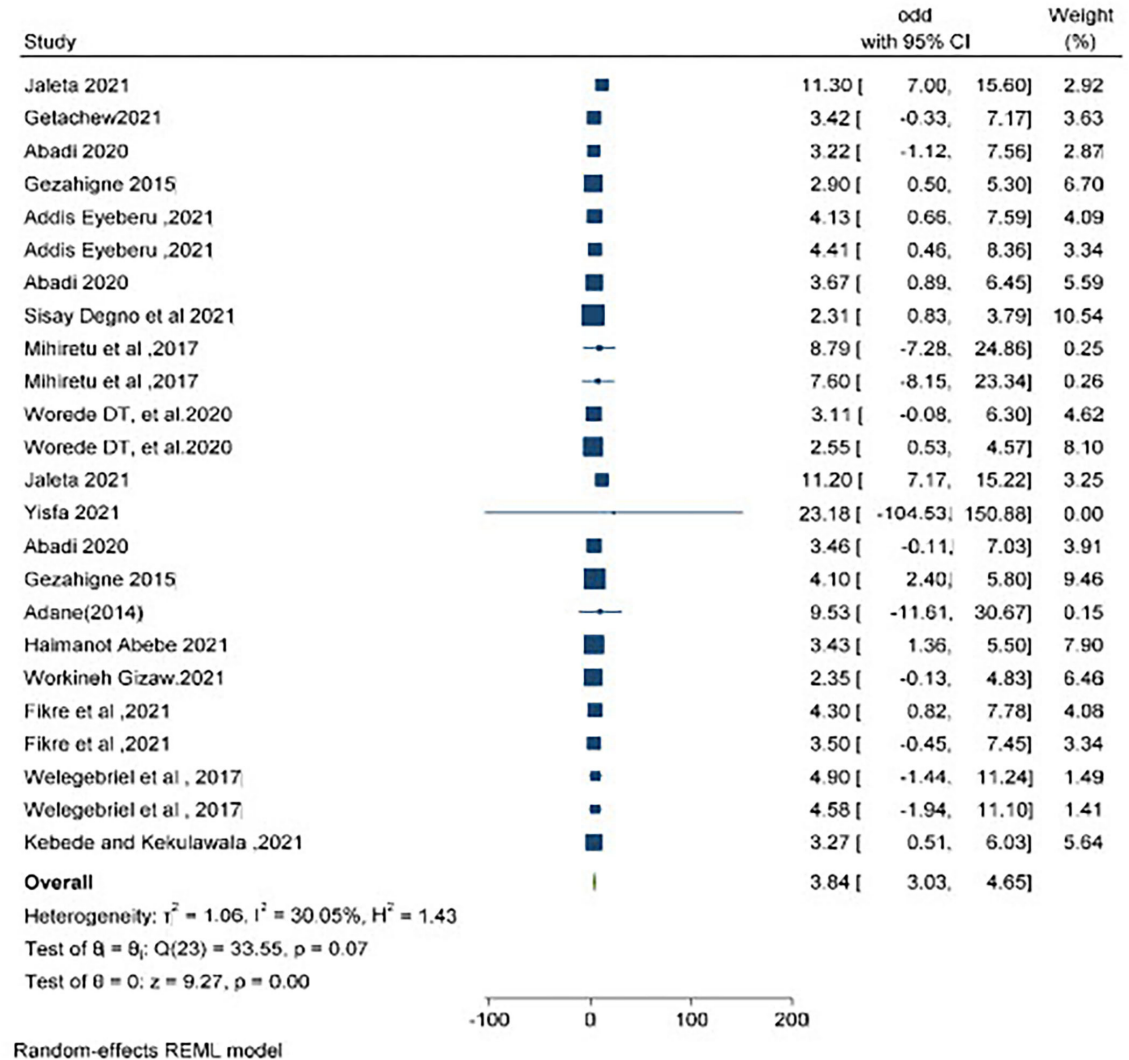
Comparison of prenatal death using CMA and STATA.

**Figure 7:**
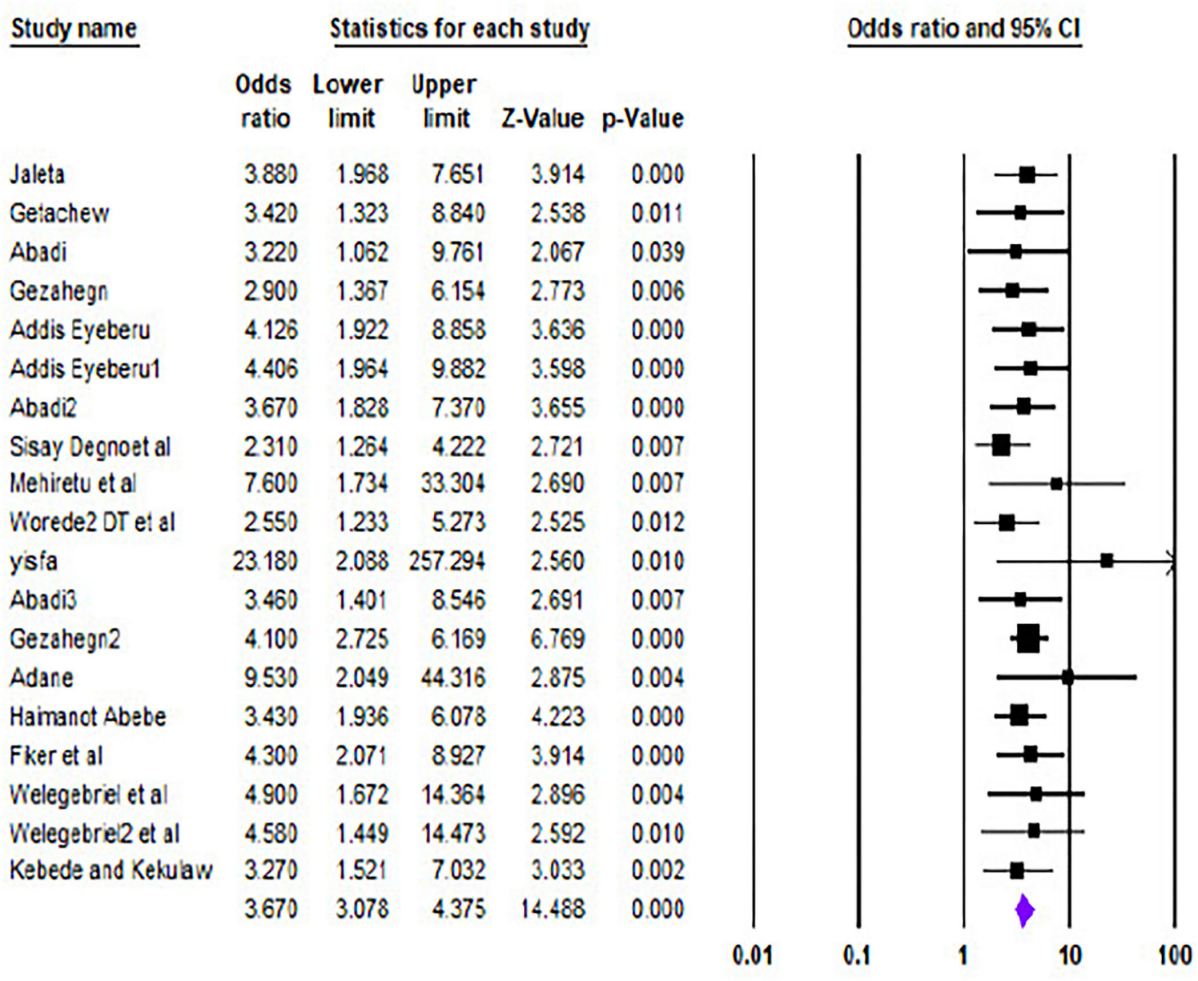
Obstetrics emergency and adverse maternal outcome.

Twelve studies described the association between obstetric emergencies and perinatal deaths. The pooled analysis showed both soft wares (i.e., STATA and CMA) (Figures 6, 7, respectively) that the likelihood of perinatal death was significantly increased by 3.84 (OR = 3.84, 95% CI 3.03–4.65) *I*^2^ = 30.05% p = 0.07) and 3.67 (OR = 3.67.95% CI 3.078–4.375) times more in a mother having obstetric emergencies than normotensive women, respectively.

### Publication bias

**Figure 8:**
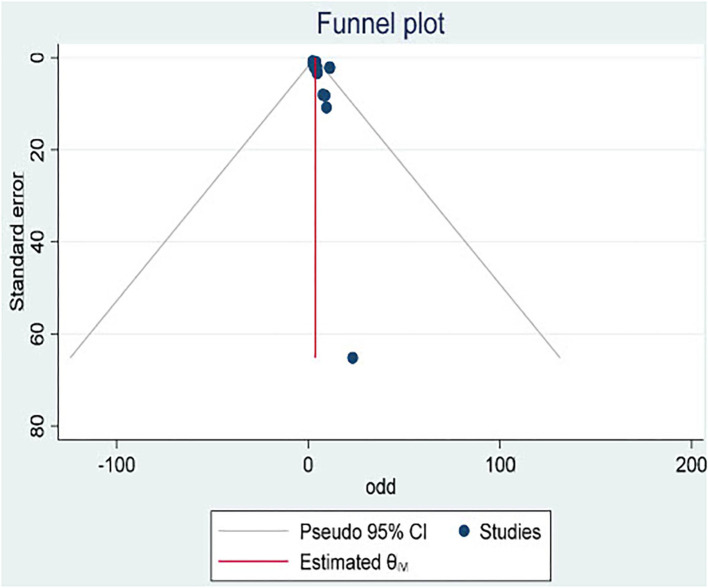
Perinatal death.

There was evidence of publication bias across studies reporting on the occurrence of perinatal death following an obstetric emergency, with Egger's test for bias giving a *p*-value of 0.014 and the asymmetry shape of the funnel plot (Figure 8).

### Sensitive analysis

Sensitivity analysis was done by removing outliers and inliers, and no significant difference was investigated. While removing outliers, we noticed that the pooled analysis revealed OR 3.69 (95% CI 3.08–4.42), and after removing inliers, the pooled analysis was OR 3.67 (95% CI 3.07–4.40), so we can say this meta-analysis result is not sensitive.

### Subgroup analysis

Subgroup analysis of perinatal death by design, region, and publication year revealed little heterogeneity (*I*^2^ = 57.41 and 20.15% based on areas) and substantial homogeneity (*I*^2^ = 0.00% based on publication year and study design)across the studies in terms of risk of perinatal death.

Subgroup analysis of prenatal death prevalence by region across Ethiopia showed a significantly higher pooled prevalence in Amhara regional state (5.89%; 95% CI = [1.64, 21.18]) and Harari regional state (4.25%; 95% CI = [2.44, 7.41] (Table 3). The subgroup analysis by study design also revealed a high pooled prevalence in retrospective study (3.92%, 95% CI = [2.86, 5.38] as well as cross-sectional (3.64%, 95% CI = [2.48, 5.33] study designs (Table 4). Subgroup analysis of prenatal death prevalence by publication year showed a significantly higher pooled prevalence than a study conducted in 2014 (9.53%; 95% CI = [2.04, 44.31]) and 2017 (5.26%; 95% CI = [2.63, 10.54]) (Table 5).

**Table 3:** Sub group analysis of obstetrics emergency based on publication year of studies in Ethiopia from 2010 to 2022.

**By pub-year**	**Pool OR 95% CI**	***I*** **^2^**
2014	9.53(2.04, 44.31)	0.00%
2015	3.78(2.64, 5.42)	0.00%
2017	5.26(2.63, 10.54)	0.00%
2020	3.17(2.11, 4.77)	0.00%
2021	3.67(3.07, 4.37)	0.00%
**By study design**	**Pool OR with 95% CI**	***I*** **^2^**
Case-control	3.52(2.61,4.74)	0.00%
Cross-sectional	3.64(2.48, 5.33)	19.38%
Prospective	3.51(2.14, 5.76)	0.00%
Retrospective	3.92(2.86, 5.38)	0.00%

**Table 4:** Obstetric emergencies and early onset of neonatal death in Ethiopia.

**No**	**Author**	**Region**	**Study design**	**Sample size**	**Type of obstetric emergency**	**Magnitude**	**OR**
1	Jaleta et al. (11)	Oromiya	Retrospective	259	Uterine rupture	10.3%	11.3(7.52,16.12)
2	Berhe et al. (36)	Tigray	Prospective	260	PIH	5%	3.22(1.06,9.74)
3	Belay Tolu et al. (37)	Addis Abeba	Prospective	164	Pre-eclampsia	1%	2.59(1.97,3.42)
4	Gezahegne (2015)	SNNPR	Retrospective	1,015	HDP	18.9%	2.9(1.37, 6.17)
5	Getachew et al. (13)	SNNPR	Unmatched case-control	207	PIH	17.4%	3.42(1.32,8.82)
6	Eyeberu et al. (41)	Harari	Cross-sectional	834	APH	5.1%	4.12 (1.92,8.85)
					PIH	4.9%	4.40 (1.96,9.86)

**Table 5:** Sub-group analysis of obstetrics emergency based on region of studies in Ethiopia from 2010 to 2022.

**By regions**	**Pool OR with 95% CI**	***I*** **^2^**
Addis Abeba	3.27(1.52,7.03)	0.00%
Amhara	5.89 (1.64, 21.18)	57.41%
Harari	4.25(2.44,7.41)	0.00%
Oromia	2.92(1.76,4.84)	20.15%
SNNPR	3.91(3.04,5.02)	0.00%
Tigray	3.51(2.14,5.76)	0.00%

The meta-regression indicates that the number of publications on obstetric emergencies is on the rise, which shows that perinatal death following obstetric emergencies is currently receiving attention (Figure 7). Our finding indicated that every unit increment of obstetric emergencies leads to the risk of perinatal death also increasing by a unit (Figure 8).

### Obstetric emergencies and adverse perinatal outcomes

**Figure 9:**
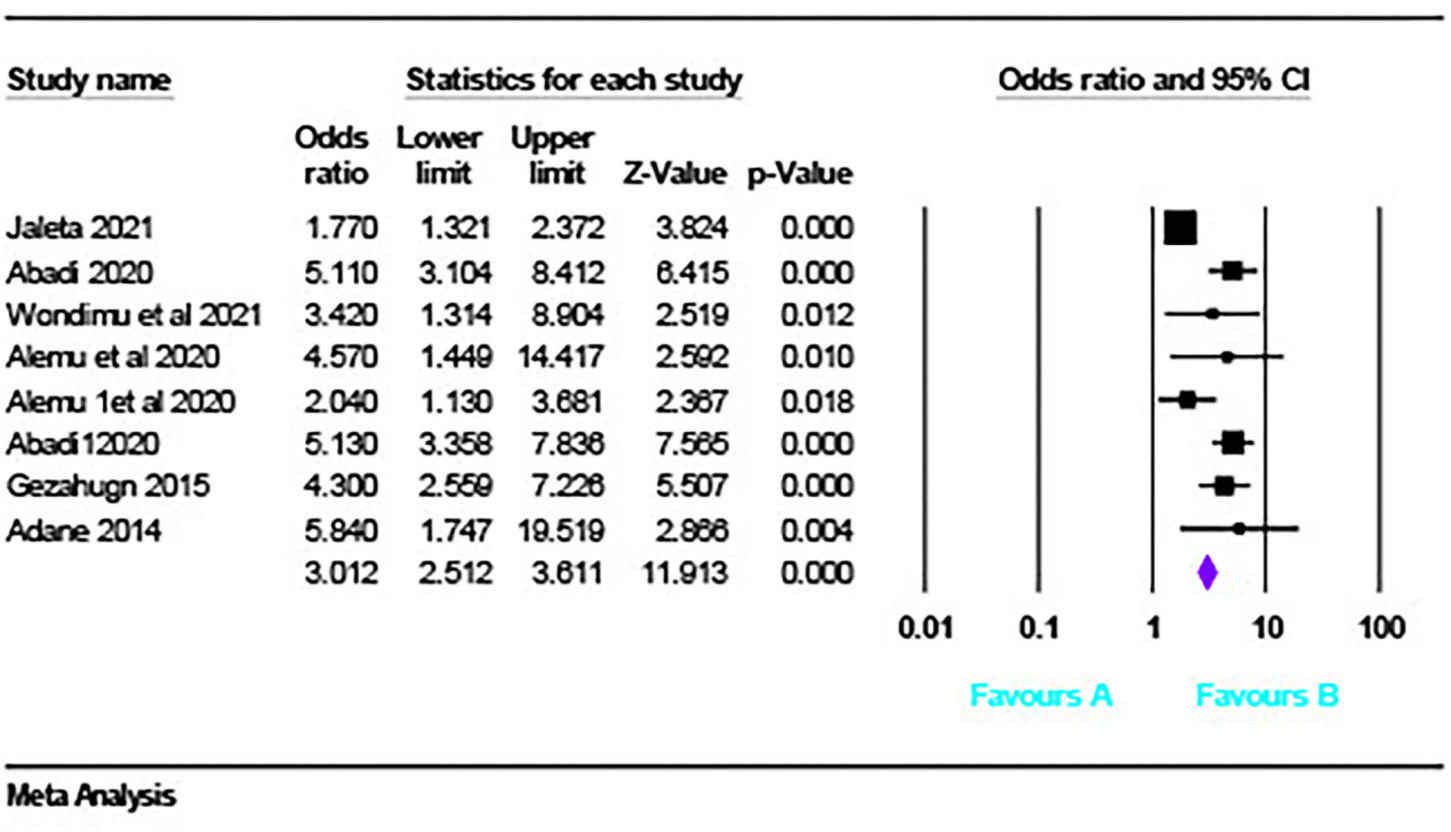
Obstetrics emergency and adverse perinatal outcome.

Six studies described the association between obstetric emergencies and adverse perinatal outcomes (LBW, NICU admission). The pooled analysis showed that the risk of adverse perinatal outcome was three times (OR = 3.012, 95% CI [2.51 – 3.61] *p* < 0.001, *I*^2 =^ 75.24%) (Figure 9) more likely to occur in women having obstetric emergencies than normotensive women.

## Discussion

Our findings demonstrated that women with obstetrical difficulties had higher maternal and perinatal morbidity and mortality, which is consistent with a study conducted in Sub-Saharan African nations but slightly higher than a survey conducted in China and the WHO multi-country prevalence. This could be attributed to poor health-seeking behavior, as women are more likely to present late and with severe diseases. This study's preterm birth rate was comparable to that of a study conducted in Sub-Saharan African countries (44%). Our findings suggested that the risk of neonatal death was higher than that suggested by Latin American adverse outcome research following Vasa-preavia (1.19–2.05%), which could be attributed to the fact that in our nation, the three delays contributed to 40% of bad pregnancy outcomes (28). The unfavorable maternal outcome following an obstetric emergency was 15.9%, according to our findings. This conclusion was more significant than a multi-country study finding of 4.31% among women with pre-eclampsia but lower than the 18.06 % among women with eclampsia.This could be related to the study area, sample size, and variance in inclusion criteria. Our findings also revealed that the adverse perinatal outcome following an obstetric emergency in Ethiopia is 37.1 %, which is higher than the global average of 30.89 % (women with pre-eclampsia). This could be due to differences in inclusion criteria (29).

### Obstetric emergencies and adverse maternal outcomes

An obstetric emergency is a potentially fatal obstetrical condition that causes considerable maternal and fetal morbidity and mortality. Furthermore, our findings revealed that maternal and neonatal morbidity and mortality were common among women who had experienced obstetrical difficulties. According to the meta-analysis, the probability of a negative maternal outcome is 2.9 times higher in mothers with an obstetric emergency than in normotensive women. This finding was in line with a study conducted in SSA, which found that pregnant women with hypertension were 3.1 times more likely than normotensive pregnant women to have a cesarean section. However, this result is lower than that of a Pakistani study, which found that women with hypertension were 6.23 times more likely to have a negative outcome. (30).This could be related to the difference in the number of papers included in the meta-analysis, which varies from 0 to 1.8 times in high-income nations. Because of variations in the incidence and quality of obstetric treatment provided to emergency obstetric patients, there is such a large disparity (31).

### Obstetric emergencies and perinatal deaths

In terms of perinatal deaths, the pooled analysis revealed that the risk of perinatal death was significantly increased by 3.84 times, and the meta-analysis revealed medium heterogeneity in women having obstetric emergencies compared to normotensive women, and this finding was lower than a study conducted in SSA, which revealed that perinatal mortality was 8.2 times more significantly associated with HDP, and the meta-analysis revealed large heterogeneity, *I*^2^ = 96.9%, *p* < 0.0001 (32). This could be due to differences in geography, sample error, and the methodology of each study.

### Obstetric emergencies and LBW

The pooled analysis of our findings showed that the risk of LBW was significantly increased by 4.77 times greater in women having obstetric emergencies than in normotensive women. This finding was almost similar to a study finding of SSA; the likelihood of LBW was 3.23 times greater in women having PIH (32).

### Obstetric emergencies and NICU admission

The pooled analysis of our findings revealed that the risk of NICU admission was five times higher in women giving birth while having obstetric emergencies than in normotensive women. Our finding was higher than a study conducted in Georgia, shows that newborns whose mothers received intensive care due to obstetric emergencies had 1.16 times increased odds of being admitted to NICU (33). This might be due to care management differences and differences in the study area.

### Strengths and weaknesses of the study

Studies were carefully chosen using a rigorous search technique to ensure that retrospective and prospective cohorts and cross-sectional studies were included unbiasedly. Despite carefully selecting relevant and well-conducted research, there was a wide range of reported obstetric emergency magnitudes. Because of the small number of studies evaluating outcomes, particularly negative maternal outcomes, it was impossible to draw firm conclusions. The research was only conducted in six regions of the country, which may have diminished its representativeness. Additionally, several included studies had small sample sizes, which may affect the estimation of the results. Most of the included studies have been predominantly conducted in tertiary health institutions, and the findings may not reflect outcomes in low-level facilities. This study hadn't addressed factors for the occurrence of obstetric emergencies.

## Conclusion

This review demonstrated the high prevalence of perinatal mortality among pregnant women with one of the obstetric emergencies in Ethiopia. Adverse maternal and perinatal outcomes following obstetric emergencies such as ICU admission, development of PPH, giving birth *via* CS, maternal death, NICU admission, LBW, and perinatal death were commonly reported in this study.

## Recommendations

Because three delays in our country (delay in diagnosis, delay in early referral, and delay in providing quality of care) contributed to 40% of adverse pregnancy outcomes, it is recommended to intervene at three levels to improve the maternal and fetal outcomes of obstetric emergencies in Ethiopia; this helps to ensure the safety of pregnant women and their babies.

## Data availability statement

The original contributions presented in the study are included in the article/supplementary material, further inquiries can be directed to the corresponding author/s.

## Author contributions

All authors listed have made a substantial, direct, and intellectual contribution to the work and approved it for publication.

## Conflict of interest

The authors declare that the research was conducted in the absence of any commercial or financial relationships that could be construed as a potential conflict of interest.

## Publisher's note

All claims expressed in this article are solely those of the authors and do not necessarily represent those of their affiliated organizations, or those of the publisher, the editors and the reviewers. Any product that may be evaluated in this article, or claim that may be made by its manufacturer, is not guaranteed or endorsed by the publisher.
